# Comparative Genomics and Phylogenomics of Novel Radiation-Resistant Bacterium *Paracoccus qomolangmaensis* sp. nov. S3-43^T^, Showing Pyrethroid Degradation

**DOI:** 10.3390/microorganisms13112441

**Published:** 2025-10-24

**Authors:** Yang Liu, Tuo Chen, Yiyang Zhang, Lu Zhang, Xiaowen Cui, Tian Cheng, Guangxiu Liu, Wei Zhang, Gaosen Zhang

**Affiliations:** 1 Key Laboratory of Cryospheric Science and Frozen Soil Engineering, Northwest Institute of Eco-Environment and Resources, Chinese Academy of Sciences, Lanzhou 730000, China; liuyang21@nieer.ac.cn (Y.L.);; 2 Key Laboratory of Extreme Environmental Microbial Resources and Engineering, Lanzhou 730000, China; 3 Key Laboratory of Ecological Safety and Sustainable Development in Arid Lands, Northwest Institute of Eco-Environment and Resources, Chinese Academy of Sciences, Lanzhou 730000, China; 4 Key Laboratory of Desert and Desertification, Northwest Institute of Eco-Environment and Resources, Chinese Academy of Sciences, Lanzhou 730000, China; 5 Gansu Academy of Aco-Environmental Sciences, Lanzhou 730020, China

**Keywords:** *Paracoccus*, Mount Everest, radiation-resistance, antioxidation, cyhalothrin degradation, comparative genomics

## Abstract

This study focused on the multifunctional characteristics and bioremediation potential of *Paracoccus* spp. A novel Gram-stain-negative, aerobic, non-motile, ellipsoidal bacterium, named *Paracoccus qomolangmaensis* S3-43^T^, was isolated from moraine samples collected from the north slope of Mount Everest at an altitude of 6109 m above sea level (a.s.l.). To clarify the phylogenetic relationship of this strain within the *Paracoccus* genus and systematically characterize its features, analyses were conducted using polyphasic taxonomy and comparative genomics. Results revealed two distinct functional characteristics of strain S3-43^T^: First, strain S3-43^T^ exhibits exceptional radiation resistance, particularly tolerance to ionizing radiation. Genome annotation indicates abundant DNA repair and antioxidant-related genes (e.g., *vsr*, *mutL*, *mutS*, *ruvC*, *radA*, *addA*, *recA*, *recN*, *recO*). Second, strain S3-43^T^ contained several pyrethroids degradation related genes, including cytochrome P450, monooxygenase, and aminopeptidase. The results of the genomic comparison of strain S3-43^T^ with related type strains also revealed differences and distribution of key genes related to stress response, environmental variables, and bioactive metabolites. Based on the results of the polyphasic taxonomic analysis, strain S3-43^T^ (=KCTC 8297^T^ = GDMCC 1.3460^T^) should be classified as a novel species of the genus *Paracoccus*, designated as *Paracoccus qomolangmaensis* sp. nov.

## 1. Introduction

Mount Everest, the core of the Earth’s Third Pole, presents an extreme “multiple stressor” environment, characterized by high altitude, strong radiation (10–20X cosmic rays sea level annual dose, intensified UV inducing DNA breaks, and protein/lipid oxidation, demanding robust DNA repair and antioxidant systems), oligotrophic (the moraine composed of glacial rock debris, sand, and fines exhibits oligotrophy, total organic carbon <0.1%), and low temperatures (annual averages of −15 °C to −10 °C, extremes to −40 °C, with >30 °C diurnal fluctuations), making it difficult for most organisms to survive there in the long term [1,2,3]. Especially at higher altitudes, the cosmic rays received there further hindered the evolution and development of life in such extremely glacial environments [2]. However, the extreme pressures of glacial environments also drive microorganisms to evolve unique adaptive strategies. Proteobacteria, as the dominant group in glacial environments [4] (including moraine, ice, and glacial meltwater [5]), exhibits significant advantages under glacial conditions. Moreover, species of Pseudomonadota, which are oligotrophic bacteria inhabiting nutrient-poor environments like deep-sea sediments, glacial ice, deep subsoil, and so on, possess well-known capabilities of antioxidation, radiation-resistance, degradation of pollutants, and production of abundant secondary metabolites with important applications [6,7,8,9,10]. Therefore, there is an urgent need to discover more stress-resistant microbial resources in glacial environments to explore their mechanisms of stress resistance and uncover their potential applications.

The genus *Paracoccus*, belonging to the phylum Pseudomonadota, was first proposed by Davis et al. [11], with *Paracoccus denitrificans* designated as the type species [12]. All species of the genus *Paracoccus* are Gram-negative. Members of this genus have been isolated from various environments, such as soil, air, plant inter-roots, horse blood, etc. To date, the genus *Paracoccus* includes 91 validly published novel species (https://lpsn.dsmz.de/genus/paracoccus, accessed on 15 May 2025); marine isolates from different habitats have the highest abundance (~34%), followed by soil isolates (~28%) [13]. Strains affiliated to *Paracoccus* isolated from oligotrophic environments are still rare. For instance, only four type strains were isolated from extremely glacial habitats [14,15,16,17,18]. Furthermore, from the perspective of molecular phylogenetics, single-gene-based phylogenies are not suitable for *Paracoccus* spp., as many species of *Paracoccus* spp. sequences with higher than 99% 16S rRNA gene similarity, but were identified as novel species based on DNA-DNA hybridization (DDH) values [13,19]; genomic metrics such as DDH, Average Nucleotide Identity (ANI), and Average Amino Acid Identity (AAI) were used to overcome the limitations based on single gene [20].

In addition, with regard to the potential applications of *Paracoccus* species, most *Paracoccus* spp. have been reported to their bioremediation potential to degrade various environmental pollutants, such as deltamethrin, lindane, carbofuran, polycyclic aromatic hydrocarbons (PAH), etc. For example, *Paracoccus* sp. YM3 can metabolize carbofuran (50 mg/L) to carbofuran-7-phenol, using the pesticide as the only source of carbon. *Paracoccus* sp. NITDBR1 can use lindane as the sole source of carbon and could degrade up to 90%, and there are also strains that can act as methylotrophs utilizing C1-carbon compounds as a substrate and accumulating polyhydroxyalkanoates (PHA) [21,22,23,24]. Due to its environmental restoration capabilities and resilience to adverse conditions, previous studies have reported that only *Paracoccus everestensis* S8-55^T^ isolated from the north slope of Mount Everest has been reported to exhibit antioxidant capacity [14]. *Paracoccus yibinensis* WLY502^T^ contains genes such as *malK*, *ugpE*, *ugpA*, *crtY*, and *ispA*, which are involved in astaxanthin production to repair oxidant damage [25].

Although the above findings indicate that *Paracoccus* spp. are potential genera for bioremediation of the environment, systematic studies of *Paracoccus* species derived from strong-irradiated glacial environments remain scarce, especially radiation-resistant species. Specifically, existing relevant studies have primarily focused on the isolation of subglobular bacteria in cold environments (non-radiation-dominated): *Paracoccus gahaiensis* CUG 00006^T^, *Paracoccus tibetensis* S9a3^T^, *Paracoccus nototheniae* I-41R45^T^, and *Paracoccus liaowanqingii* 2251^T^ were isolated from Gahai Lake and permafrost of Qinghai-Tibetan Plateau, black rock cod fish from the Chilean Antarctic, and Tibetan antelope [15,16,17,18]. Although these strains are adapted to low temperatures or oligotrophic conditions, the radiation intensity in their habitats has not been explicitly correlated. Only Cui Et Al. isolated *Paracoccus everestensis* S8-55^T^ with antioxidant capability from the north slope of Mount Everest [15]. However, this study did not delve into its radiation resistance mechanism nor address its pollutant degradation function. As mentioned, low-temperature glacial environments typically receive intense solar radiation, especially high-altitude mountain glaciers [1,2]. Microorganisms surviving under such environmental stress may evolve multifunctional adaptive mechanisms that combine cold tolerance, radiation resistance, and efficient nutrient utilization. Therefore, radiation-resistant strain resources and potential applications of *Paracoccus* species derived from glacier environments require further exploration and investigation. The purpose of this study is to describe a novel species in the genus *Paracoccus* and to show some of the species’ capabilities via polyphasic taxonomic methods, and by combining genome sequencing with comparative genomic analysis, elucidate the molecular mechanisms of radiation resistance and pyrethroids degradation capacity of strain S3-43^T^. The findings not only expand our understanding of *Paracoccus* species diversity and adaptive strategies in extreme cryogenic and highly irradiated environments, but also provide valuable genomic resources for studying radiation resistance mechanisms and developing novel pollutant degradation processes.

## 2. Materials and Methods

### 2.1. Isolation, Identification, and Growth Conditions

Moraine samples were collected near the East Rongbuk Glacier on the northern slope of Mount Everest in the Tibet Autonomous Region of China (28.02° N, 86.56° E). Samples were collected in May 2022 at a depth of 0~5 cm, stored at −20 °C in Labplas sterile bags (EFR-5590E, TWIRL’EM^®^, Labplas Inc., Montreal, QC, Canada). Strain was isolated from moraine on Reasoner’s 2A (R2A) agar [26]. Take 5 g of moraine sample with 20 mL of sterile saline (0.85%, *m*/*v*) into a solution 30 °C, 200 rpm shaking for 40 min, stand for 2 min, then dilute the supernatant to a concentration of one part per thousand, and then 200 μL of the supernatant was pipetted and spread on R2A agar medium. After 15 days of incubation at 30 °C, strain S3-43^T^ was isolated and purified on R2A agar medium. Strain was deposited in the Guangdong Microbial Culture Collection Center (GDMCC) and Japan Collection of Microorganisms (JCM). Using universal bacterial primers 27F and 1492R, the complete 16S rRNA gene was amplified by Polymerase Chain Reaction (PCR) [27]. Upon receiving the purified PCR products, Tsingke Company (Xi’an, China) performed bidirectional sequencing using the dideoxy chain termination method with ABI 3730XL analyzer (Applied Biosystems v1.3.1, Waltham, MA, USA). Sequences were compiled using SeqMan software (Lasergen v18.0.3, Houston, TX, USA). The 16S rRNA gene sequence database in EzBioCloud performs 16S rRNA sequence alignment using the BLAST method, matching and comparing with the most closely related type strain [28]. The 16S rRNA sequence of strain S3-43^T^ was deposited in the GenBank database with DDBJ/EMBL/GenBank accession number ON527558. Phylogenetic trees for the 16S rRNA sequences, including the 41 closest type strains of the genus *Paracoccus*, with *Pikeienue piscinae* RR4-56^T^ used as an outgroup, were reconstructed using the neighbor-joining [29], minimum-evolution [30] and maximum-likelihood [31] method in Mega V11.0 [32], and topologies of the resultant trees were calculated by bootstrap analysis according to 1000 resamplings [31].

### 2.2. Morphological, Physiological, Biochemical, and Chemotaxonomic Analysis

Multiple identifications were performed on the strains. The cell morphology of strain S3-43^T^ was observed [33], and cells were subjected to Gram staining using the Solarbio Gram-Stain Kit (Solarbio, Beijing, China). Experimental measurements were also conducted to determine the tolerance of strain S3-43^T^ to temperature (10, 20, 30, 35, 40, and 45 °C), pH (pH 4.0–12.0, interval 1), and NaCl (0–10%, *w*/*v*, interval 1%). The methods of carbohydrate and nitrogen utilization tests were performed according to Shirling et al. and Williams et al. [34,35]. Using the method of Kurup and Schmitt, we determined the production of catalase and oxidase and evaluated the hydrolysis of Tween 20, Tween 80, starch, and gelatin [36]. Additional physiological and biochemical characteristics were analyzed for cells cultured at 30 °C. The assays were performed using API ZYM and API 20NE test strips (bioMérieux, Craponne, France) in accordance with the manufacturer’s protocols. The whole-cell sugar composition [37] and polar lipid pattern [38] of strain S3-43^T^ were detected by TLC. Cellular fatty acids were extracted, methylated, and identified using Sherlock MIDI (Newark, DE, USA, Microbial Identification System 6.2b) and a gas chromatograph (6890N; Agilent, Santa Clara, CA, USA), followed by peak identification via the TSBA 6 (version 6.21) database [39]. The analysis method of menaquinones was described by Collins et al. [40].

### 2.3. Whole Genome Sequencing, Assembly, and Annotation

We extracted genomic DNA from 72h aerobic cultures grown in R2A liquid medium at 30 °C and 160 rpm, using the Bacterial Genomic DNA Extraction Kit (Omega, Norcross, GA, USA), following the manufacturer’s recommendations. The genomic DNA extracted from strain S3-43^T^ was paired-end sequenced on an Illumina HiSeq2000 (Illumina, Inc., San Diego, CA, USA). The genome was assembled de novo by the Velvet 1.2.10 program [41,42]. Sequencing depth was 100× to use high-quality datasets for analysis. Genome assembly was conducted in two stages: the draft genome was generated with SOAPdenovo2 [41], and the complete genome was finalized using Unicycler v0.4.8 [42]. Subsequently, protein-coding genes were predicted from the complete chromosomal assemblies with Glimmer 3.02 (http://ccb.jhu.edu/software/glimmer/index.shtml accessed on 10 May 2025). The NCBI prokaryotic genome annotation pipeline (PGAP) [43] was used to predict tRNA genes, rRNA genes, and non-coding rRNA genes. Genome annotation was carried out employing the RAST system (Rapid Annotation using Subsystem Technology) [44]. The UBCG phylogenomic tree was reconstructed based on the genome using the UBCG pipeline (Up-to-Date Bacterial Core Gene set and pipeline) for 92 core genes [45]. A total of 68 genomes from the *Paracoccus* type strains were retrieved from the NCBI database on 22 July 2024.

### 2.4. Radiation Resistance Analysis

The radiation resistance of strain S3-43^T^ was assessed. We used the reference type strains *Deinococcus radiodurans* R1^T^ as the positive control and *Escherichia coli* BL 21^T^ as the negative control. Strains S3-43^T^, R1^T^, and BL21^T^ were evaluated for resistance to UV-C irradiation, γ-ray irradiation, heavy-ion irradiation, and antioxidant. Strains S3-43^T^, R1^T^, and BL21^T^ were inoculated into R2A liquid medium and cultured to the logarithmic growth phase (OD_600_ = 1). The bacterial suspensions were then diluted to a concentration of 10^−4^ relative to the original concentration. Take 100 μL of strain dilutions and spread it on R2A agar medium and irradiate it with UV-C irradiation at 0, 50, 100 J/m^2^, incubated for 7–14 days, and counted. Take 20 mL of the original solution and irradiate it with γ-rays using a Co-60 source γ-ray meter (Qingdao, China, Zhongke Tongxing Nuclear Technology Co., Ltd.) at 0, 50, and 100 kGy. Take 1 mL of diluted solution in a 3 cm diameter Petri dish and subject it to 12C^6+^ ion beam irradiation at 0, 250, and 500 Gy. (The 12C^6+^ ion beam irradiation parameters are as follows: after passing through a 50 μm stainless steel window, a 20 μm Mylar film, and a 1.3 m of air gap, the energy of the 12C^6+^ ions from 80 MeV/u decays to 76.37 MeV/u; the range in the water was expected to be 16 mm, and the peak position was 15.5 mm.) After γ-rays and ion beam irradiation, 100 μL of irradiated bacterial suspension was coated on R2A agar medium, incubated for 7–14 days, and counted. The colonies of the irradiated plates were counted as N; the colonies of the unirradiated plates were counted as N_0_, and the survival rate of the irradiated strains was calculated as SR (Survival Rate) = N/N_0_ × 100% to determine the strength of the strain’s resistance to radiation [7]. All tests had three replicates.

### 2.5. Antioxidant Analysis

The antioxidant capacity of the strains was evaluated by assessing their viability in the presence of H_2_O_2_, a weak oxidant that permeates cell membranes and induces intracellular damage [46]. We evaluated the antioxidant capacity based on the survival rate of the strain after exposure to hydrogen peroxide. Use the reference type strains *Deinococcus radiodurans* R1^T^ as the positive control and *Escherichia coli* BL 21^T^ as the negative control. Strains were cultured in R2A liquid medium, and the bacterial suspension (OD_600_ = 1) was mixed with H_2_O_2_ solution to make the final mixed solution of hydrogen peroxide concentration, which was 0–100 mM at intervals of 10 mM. Subsequently, the centrifuge tubes were incubated for four hours at 30 °C under agitation (160 rpm). Diluted the mixed solution into 10,000 dilutions with 0.9% normal saline, inoculated 100 μL diluent on R2A agar medium, cultured it in a constant temperature incubator at 30 °C for 7 days, and counted. The colonies of the treated plates were counted as N; the colonies of the untreated plates were counted as N_0_, and the survival rate of the strains under oxidative stress was calculated as SR (Survival Rate) = N/N_0_ × 100% to determine the strength of the strain’s resistance. All tests had three replicates.

### 2.6. Cyhalothrin Degradation Analysis

The cyhalothrin degradation experiment was carried out in 500 mL flasks containing 150 mL of sterile R2A liquid medium containing 50 mg/L cyhalothrin. The strain was inoculated into the medium and incubated in a shaker at 30 °C, 180 rpm for 5 days, and the concentration of residual cyhalothrin was measured by high performance liquid chromatography (HPLC) every 12 h. The growth of the strain was determined by OD_600_. All tests had three replicates.

### 2.7. Comparative Genomic Analysis

Genomic data of species related to strains S3-43^T^ were obtained from GenBank to evaluate their similarity. We computed the Average Nucleotide Identity (ANI) with the OrthoANIu (OrthoANI), BLAST (ANIb), and MUMmer (ANIm) algorithms [28,47,48,49]. We computed the digital DNA–DNA hybridization (dDDH) values using the GGDC 3.0 tool [50]. Specifically, the values were derived from the recommended formula 2, chosen for its independence from genome length and robustness when handling incomplete draft genomes. We computed the Average Amino acid Identity (AAI) with the online tool developed by the Konstantinidis group (http://enve-omics.ce.gatech.edu/aai/ accessed on 10 May 2025) [51]. KEGG (Kyoto Encyclopedia of Genes and Genomes) [52], COG (Clusters of Orthologous Groups of proteins) [53], NR (NCBI Non-Redundant Protein) [54], Pfam (Protein Families) [55], Swiss-Prot [56], and CAZy (Carbohydrate Active Enzymes) [57] databases were selected for retrieval to improve the general functional annotation. The biosynthetic gene cluster of secondary metabolites was predicted by silicon calculation using AntiSMASH 7.0 [58]. Pan-genomes were analyzed using PGAweb [59].

### 2.8. Data Analysis and Statistics

All experiments were performed in triplicate. The phylogenetic tree was constructed by MEGA 11.0. Statistical analysis was carried out using SPSS 16.0 and R 4.1.0 software. Data analysis and graphical representation were conducted with OriginPro 2022. Adobe illustrator 2020 was used to draw figures.

## 3. Results and Discussion

### 3.1. Phenotypic, Physiological, Phylogenetic, and Phylogenomic Characteristics

**Figure 1:**
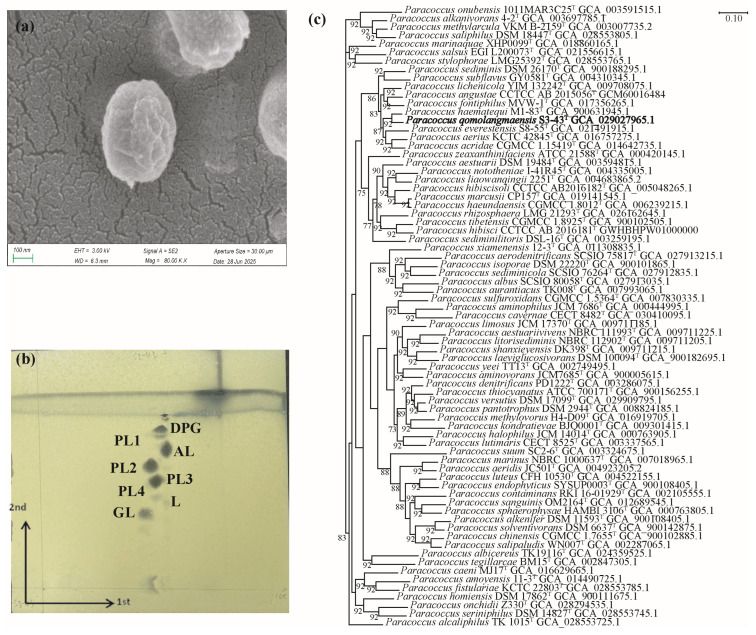
Chart of basic properties of strain S3-43^T^. (**a**) Scanning electron microscope photo of strain S3-43^T^, (**b**) Polar lipids profile of strain S3-43^T^, and (**c**) UBCG phylogenetic tree based on the Up-to-Date Core Gene set and pipeline of strains S3-43^T,^ and strains of other 69 species in the genus *Paracoccus*. Phylogenetic tree generated with UBCG using the amino acid sequences. The number at the nodes indicates the gene support index. The numbers at the nodes indicate the gene support index. Bar, 0.10 substitutions per nucleotide position.

The colonies of strain S3-43^T^ cultured in R2A medium after 72h were milky white and spherical. The cells of S3-43^T^ were aerobic, Gram-negative, and ellipsoid (0.6–0.9 μm × 0.7–1.1 μm) (Figure 1a), without spore formation and motility. The strain grew with the optimal temperature of 30 °C, the optimal pH = 8.0, and without NaCl. Strain S3-43^T^ was positive for oxidase activity, reduction in nitrate, and alkaline phosphatase, but negative for esterase lipase (C8) and N-acetyl-β-glucosaminidase. Strain S3-43^T^ could not use L-Arabinose or D-xylose as the sole carbon source. The different physiological characteristics of S3-43^T^ and its reference strains are shown in Table S1. The physiological characteristics distinguish strain S3-43^T^ from its closest type species in the genus *Paracoccus*. The chemotaxonomic characteristics such as major fatty acids, predominant respiratory menaquinone, and polar lipids of strain S3-43^T^ correspond to those of other members of the genus *Paracoccus* (Table S2, Figure 1b and Figure S4).

Pairwise 16S rRNA gene sequence comparisons showed that strain S3-43^T^ has the highest pairwise similarity of 98.76% with *P. haematequi* M1-83^T^. The 16S rRNA-based neighbor-joining, minimum-evolution, maximum-likelihood phylogenetic trees (Figures S1–S3) and the core gene-based UBCG phylogenomic tree collectively determined the placement of strain S3-43^T^ within the genus *Paracoccus* cluster (Figure 1c). The assembled genome sequence of strain S3-43^T^ (DDBJ/EMBL/GenBank accession number CP119082) encodes 3746 CDS, 46 tRNAs, and two copies of the 16S rRNA gene. Genomic characterization of strain S3-43^T^ is similar to other species of the genus *Paraucoccus*. Detailed genomic information on the genome sequences of the reference strains is provided in Table S3. ANIb, ANIm, and OrthoANI evaluations indicate that the overall genome relatedness between the strain S3-43^T^ (Figure S5) and its closest relatives is below the 95% cut-off value for grouping genomes of the same species [60]. The genome sequences of strain S3-43^T^ and related species had the highest dDDH value of 49.10% (*P. haematequi* M1-83^T^). The results of the dDDH analysis of the strain S3-43^T^ and its closest neighbors were below the threshold of 70% established for delineating prokaryotic species (Figure S5) [61]. The highest AAI value between S3-43^T^ and related species was 91.91% (Figure S5), which is also lower than 95%, the threshold defined by prokaryotic species [62]. These suggest that strain S3-43^T^ represents a novel species of the genus *Paracoccus*.

Based on the presented results for phylogenomic, genome, biochemical, and physiological analyses, strain S3-43^T^ appears to be a novel species of the genus *Paracoccus*, for which the name *Paracoccus qomolangaensis* sp. nov. is proposed.

### 3.2. The Radiation Resistance and Antioxidant of S3-43^T^

**Figure 2:**
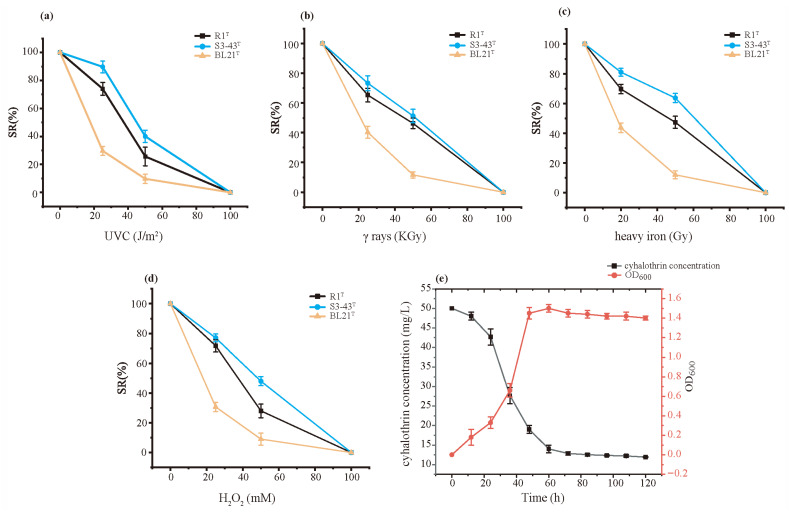
The radiation resistance, antioxidant, and cyhalothrin degradation of S3-43^T^. (**a**) Survival rates of strains after exposure to different doses of UV- C radiation, (**b**) Survival rates of strains after exposure to different doses of γ-rays, (**c**) Survival rates of strains after exposure to different doses of heavy-ion beam, (**d**) Survival rates of strains after treated with H_2_O_2_, and (**e**) Cyhalothrin degradation rate of strain S3-43^T^.

Figure 2 shows the radiation resistance and antioxidant activity of strain S3-43^T^ compared to the control strains *D. radiodurans* R1^T^ and *E. coli* BL21^T^. The radiation resistance of strain S3-43^T^ was evaluated through non-ionizing radiation (UV-C 254nm) and ionizing radiation (γ-rays and heavy-ion beam). The results show that the radiation resistance of strain S3-43^T^ is generally higher than that of the positive control *D. radiodurans* R1^T^, performing particularly well when exposed to UV-C irradiation. At the radiation dose of 25 J/m^2^, the survival rate of strain S3-43^T^ reached 89.67%, while the survival rate of *D. radiodurans* R1^T^ only reached 74.00%. Under γ-ray irradiation, the advantage of strain S3-43^T^ did not exist. However, under heavy-ion beam irradiation at a dose of 100 Gy, the survival rate of strain S3-43^T^ reached 81.00%, which is higher than that of strain *D. radiodurans* R1^T^. In addition, the antioxidant capacity of strain S3-43^T^ was assessed, showing great antioxidant ability with a slower decline in survival rate (Figure 2d). When the UV-C dose was 100 J/m^2^, the γ-ray dose was 100 KGy, the heavy-ion dose was 500 Gy, and the hydrogen peroxide concentration was 100 mM, no strain survived. This indicated that even if there were anti-radiation and antioxidant genes in the strains that expressed gene repair function, excessive UV-C radiation and hydrogen peroxide concentration would also cause cell damage.

### 3.3. Cyhalothrin Degradation Capability

Figure 2e shows the growth of strain S3-43^T^ under 50 mg/L cyhalothrin conditions and the degradation process of cyhalothrin. In the first 0–24 h, bacterial cell growth was slow, and the cyhalothrin concentration remained virtually unchanged. In the logarithmic growth phase (1–2 days), both bacterial cell numbers and rates of cyhalothrin biodegradation showed a fast upward trend, reaching a biodegradation rate of 62% by 48 h. After 60 h of incubation, bacterial cell numbers reached a peak and then gradually decreased. After 5 days, the degradation rates of cyhalothrin reached 76.2%. In conclusion, strain S3-43^T^ can effectively degrade cyhalothrin.

### 3.4. Genomic Insights into a Novel Species Related to Biological Functions

A series of comparative genomic analyses was performed to further characterize *Paracoccus qomolangaensis* S3-43^T^ and its closely related type strains at the genomic level.

#### 3.4.1. Pan-Genome Analysis

**Figure 3:**
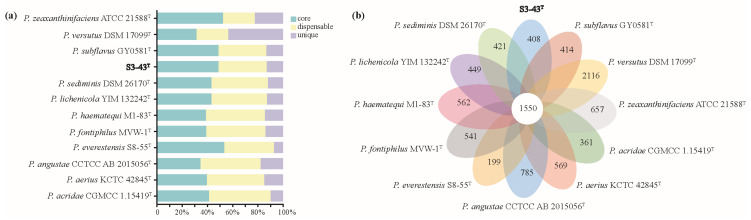
Comparative analysis of orthologous protein groups in S3-43^T^ and related 11 *Paracoccus* genomes. (**a**) Percentage of core, dispensable, and unique gene clusters in each of the 12 genomes. (**b**) Venn diagram displaying the number of core and unique gene clusters for each of S3-43^T^ and related type strains.

The pan-genome represents the complete genetic makeup of a species, serving as the gene pool of all strains within that species. It comprises core genes (homologous gene clusters/genes present in all samples), dispensable genes (homologous gene clusters/genes coexisting in two or more samples), and unique genes (homologous gene clusters/genes present in only a single sample). Bacterial pangenomes can be categorized into open pan-genome and closed pan-genome. It was shown that the genus *Paracoccus* supports an open pan-genome [20]. The 12 strains of genus *Paracoccus* in this study showed an open pan-genome based on a power-law regression function (b = 0.646, which is in the range of 0 < b > 1) (Figure S5), in which each *Paracoccus* sp. contains several unique genes, and as the genomes increase, the pan-genome pool increases further. The core size accumulation curve reached saturation after two genomes, indicating the existence of a stable minimum core. The 3365 CDS present in the genome of strain S3-43^T^ were divided into 1958 homologous gene clusters (Figure 3b), most of these gene clusters were present in the core genes (48.96%), compared to the dispensable genes (1208 gene clusters, 38.16%) and unique genes (408 gene clusters, 12.89%) genome (Figure 3a). We compared the homologous genes between strain S3-43^T^ and its closely related strains and showed that 1550 core gene clusters were shared among the genomes; the percentage range of core genes for each *Paracoccus* is between 37.71% and 53.54%. Of the 18,876 genes identified in the 1550 homologous gene clusters shared by the genomes, 17,718 genes have COG annotations, including repair genes such as superoxide dismutase, recombinant DNA repair gene *recR*, *recO*, *recA*, and *radA*, and DNA mismatch repair gene *mutS* and *mutL* (Table 1). This suggests that *Paracoccus* spp. have the potential for DNA repair. The percentage of the dispensable gene ranged from 25.00 to 48.68% among 12 genomes of *Paracoccus*. The unique genes had a wide percentage range from 6.87 to 43.28% (Figure 3a). These results underscore the high adaptability of *Paracoccus* genomes and the non-conservative nature of their gene counts. Strain S3-43^T^ has 408 unique gene clusters out of a total of 1958 clusters, and of the 423 genes identified in these unique clusters, 243 genes have COG annotations, including antioxidant genes and DNA repair genes, such as the organic hydrogen peroxide reductase OsmC/OhrA, site-specific recombinant DNA repair gene *xerD*, etc. This suggests that strain S3-43^T^ exhibited a superior capacity for DNA repair in the genus *Paracoccus*.

**Figure 4:**
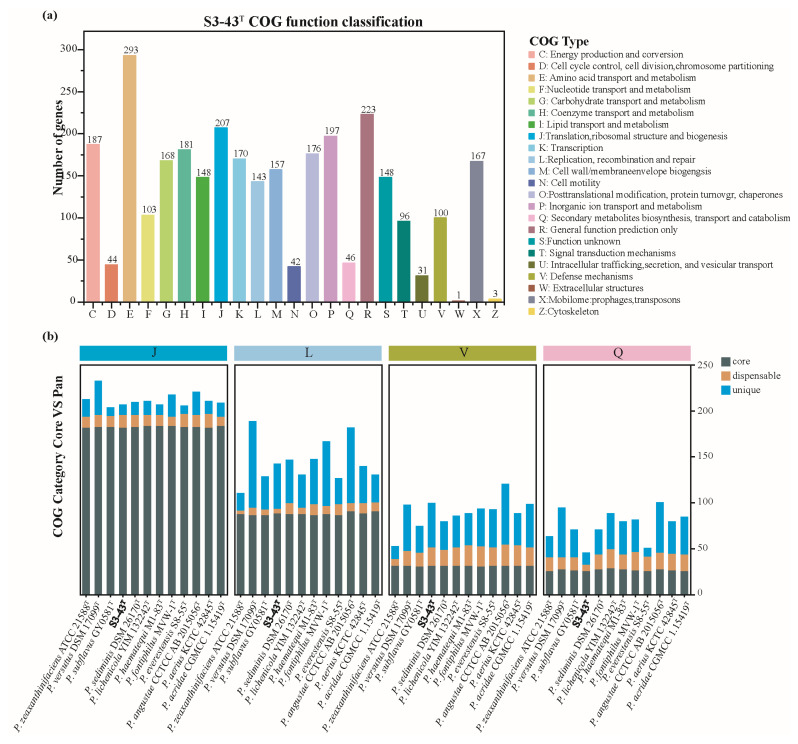
COG functional categories of strains S3-43^T^ (**a**) and COG category core vs. pan (**b**). (**a**) The horizontal axis represents different COG types, while the vertical axis represents the number of genes. For detailed functional descriptions of each COG type, please refer to the legend on the right. (**b**) The horizontal axis represents different strains and functional names at various taxonomic levels. The vertical axis indicates gene counts. Different colors within the bars denote distinct pangenome classifications.

The Clusters of Orthologous Genes (COG) database was used to study conserved protein functions among different species, reveal their evolutionary relationships, and compare species by COG functional categories [63]. The proteins that make up each homologous gene cluster are assumed to come from an ancestral protein and have the same function [64]. A total of 2761 CDS of strain S3-43^T^ (82.05% of all) were distributed in 23 COG functional categories (Figure 4a). The major functional category includes genes containing amino acid transport and metabolism (COG-E, 293 genes), general function prediction only (COG-R, 223 genes), translation, ribosomal structure and biogenesis (COG-J, 207 genes), inorganic ion transport and metabolism (COG-P, 197 genes), energy production and conversion (COG-C, 187 genes), coenzyme transport and metabolism (COG-H, 181 genes), posttranslational modification, protein turnover, chaperones (COG-O, 176 genes), transcription (COG-K, 170 genes), carbohydrate transport and metabolism (COG-G, 168 genes), mobilome: prophages, transposons (COG-X, 167 genes), cell wall/membrane/envelope biogenesis (COG-M, 157 genes), lipid transport and metabolism (COG-I, 148 genes), function unknown (COG-S, 148 genes), replication, recombination and repair (COG-L, 143 genes), nucleotide transport and metabolism (COG-F, 103 genes), and defense mechanisms (COG-V, 100 genes). Analysis of the pan-genomes of 12 strains of genus *Paracoccus* showed broadly similar functional categories related to radiation resistance and antioxidant (Figure 4b).

#### 3.4.2. Genes Potentially Associated with Radiation Resistance and Antioxidant Capabilities

As mentioned, it is speculated that strain S3-43^T^ has the potential for radiation resistance and antioxidant as isolated from the northern slope of Mount Everest were with strong radiation. Radiation damage to bacteria is mainly divided into direct and indirect damage; high doses of ionizing radiation can directly damage DNA, causing single-strand breaks (SSBs) and double-strand breaks (DSBs), with SSBs occurring 40 times more than DSBs [65]. Usually, direct damage accounts for only 20% of the cellular damage caused by radiation and 80% of the cellular damage comes from indirect damage due to oxidative stress [66,67]. Research has shown that exposure to ionizing radiation causes severe oxidative stress to all macromolecules within cells [65]. Oxidative stress refers to cellular damage caused by elevated levels of intracellular oxygen radicals and accumulation of reactive oxygen species (ROS) [68]. The accumulation of ROS arises from the radiolytic decomposition of water, including hydroxyl radicals (HO), superoxide (O_2_^−^), and hydrogen peroxide (H_2_O_2_), leading to cellular oxidative stress and damage to proteins, lipids, nucleic acids, and carbohydrates [67,69]. Bacteria protect themselves from oxidative damage by turning on the antioxidant system and DNA repair system. Oxidative stress causes DNA damage, including oxidative damage to bases and sugar-phosphates, and single or double-strand breaks (SSB or DSB) in DNA [70]. ROS react with nitrogenous bases and deoxyribose to cause oxidative reactions leading to DNA base alterations or double helix breaks [71]. ROS also causes lipid peroxidation, where the free radicals oxidize unsaturated lipid strands, leading to the formation of hydroperoxyl lipids and alkyl radicals [72].

**Figure 5:**
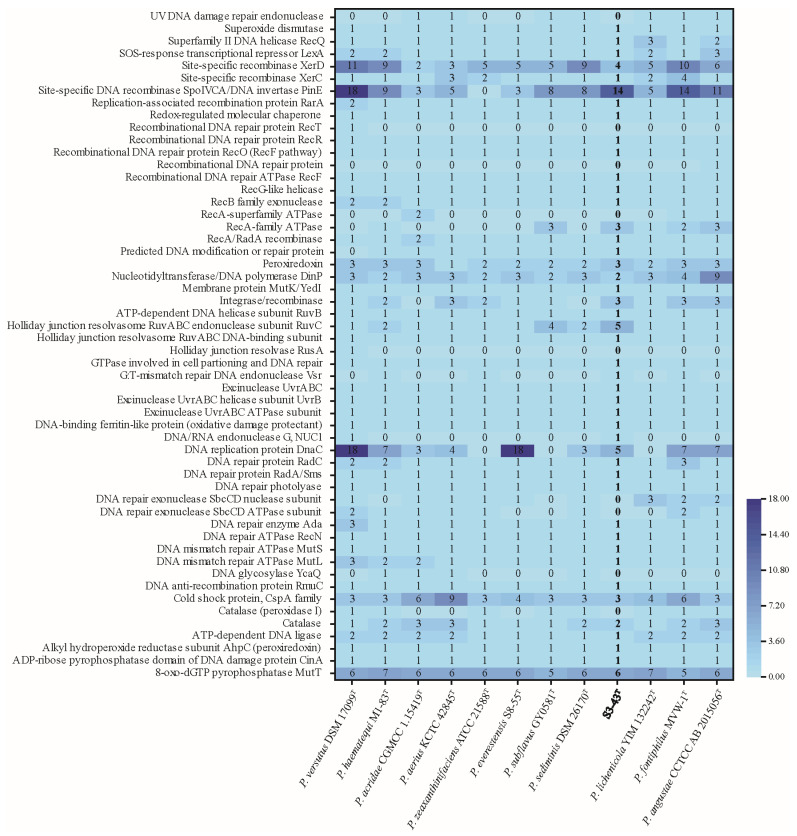
Gene lists potentially associated with radiation resistance and antioxidant activity of *Paracoccus* spp. The horizontal axis represents different strains, while the vertical axis represents the number of functional genes.

The strains in this study have a series of DNA repair genes to respond to oxidative stress-induced DNA damage (Figure 5). For example, the most common base damage caused by oxidative stress is 8-oxoguanine [70], which is mainly repaired by the base excision repair pathway (BER) initiated by the DNA N-glycosidases Fpg and OGG1, respectively, and is ameliorated by the involvement of MutT in the “GO repair system” [73,74]. In bacteria, UvrA and UvrB proteins encoded by genes *uvrA* and *uvrB* recognize and cleave damaged DNA in a multistep reaction to complete the nucleotide excision repair (NER) process [75] in response to DNA damage induced by radiation stress and oxidative stress. The results show that genes involved in antioxidant and DNA restoration described in previous studies were located in the strain S3-43^T^ genome. The genomes of 12 *Paracoccus* spp. strains were annotated to *ahpC* (peroxiredoxin), catalase, peroxiredoxin, and SOD (superoxide dismutase), constituting the enzymatic antioxidant system (Figure 5). Gene *ahpC* can encode a key antioxidant enzyme belonging to the thiol peroxidases (peroxiredoxins) family that scavenges micromolar concentrations of H_2_O_2_. Peroxidase as AhpC can be activated when millimolar concentrations of H_2_O_2_ are saturated [76,77]. SOD scavenges superoxide radicals, which generate less reactive hydrogen peroxide and oxygen with two superoxide radicals and two protons. Hydrogen peroxide is, in turn, converted to water and oxygen by catalase [70].

The possession of these antioxidant enzymes by strain S3-43^T^ is comparable to that of the other 11 strains, and it is hypothesized that *Paracoccus* spp. have a stable enzymatic antioxidant system. Meanwhile, KEGG metabolic pathway analysis showed that strain S3-43^T^ had 30 genes involved in glutathione metabolism, including *pepN*, *gshA*, *gshB*, and *gst*, suggesting that strain S3-43^T^ may metabolize glutathione as a non-enzymatic antioxidant to scavenge ROS [78]. The gene *crtB* of strain S3-43^T^ is involved in the carotenoid biosynthesis pathway, and carotenoids, as antioxidants, can greatly inhibit the effects of oxidative stress on redox status [79]. The other 11 related strains have more genes involved in the carotenoid biosynthesis pathway (gene numbers: 1–7); thus, it is hypothesized that strain S3-43^T^ does not mainly rely on carotenoid antioxidants in response to oxidative stress. A complete antioxidant system gives the strain S3-43^T^ excellent resistance to ionizing radiation.

#### 3.4.3. Gene Potentially Associated with Cyhalothrin Degradation Capability

Although pyrethroids are considered safer than other insecticides, their persistence in the environment poses high risks to non-target organisms and humans. Humans can be exposed to pyrethroid insecticides through direct skin contact and inhalation [80]. Many studies have confirmed that microorganisms can degrade pyrethroids directly by using them as carbon sources or through co-metabolism [81]. The cleavage of ester bonds is generally considered the main step in the degradation pathway of pyrethroids [82]. In the process of microbial degradation of pyrethroids, various enzymes responsible for ester bond hydrolysis (carboxylesterase, monooxygenase, and aminopeptidase) have been purified and characterized [83,84,85,86,87]. Previous research has shown that the metabolic resistance of pyrethroids is mainly related to cytochrome P450 monooxygenases (P450s), followed by carboxylesterases (CarEs) [88,89]. The biodegradation of cyhalothrin in Cunninghamella elegans is catalyzed by the monooxygenase cytochrome P450 [85]. The resistance to λ—cypermethrin in the *Helicoverpa armigera* SYR of cotton bollworm in Shenyang, China, is due to metabolic enhancement caused by overexpression of multiple P450 enzymes [90]. Heterologously expressed P450 enzymes can also metabolize pyrethroid insecticides in cotton bollworms [91]. The results indicate that the genes involved in cyhalothrin degradation described in previous studies are located in the genome of strain S3-43^T^. It is hypothesized that cytochrome P450 may be involved in the metabolism of cyhalothrin by strain S3-43^T^, catalyzing the hydrolysis of ester bonds. Strain S3-43^T^ possessed cytochrome P450 comparable to the other 11 strains (Table 2). In addition, the genome of strain S3-43^T^ was annotated with various monooxygenases and aminopeptidases, which are hypothesized to play a role in cyhalothrin degradation. Among these twelve genomes, aminopeptidase FrvX was annotated only in *P. haematequi*, *P. acridae*, *P. aerius*, and S3-43^T^, suggesting a greater potential for pyrethroids degradation by strain S3-43^T^. Strain S3-43^T^ was isolated from the northern slope of Mount Everest and has good adaptability to cold environments. Therefore, its enzymes can maintain good activity in cold environments, which is helpful for the treatment of vegetables and fruits contaminated with pyrethroids in subsequent applications. Therefore, further functional expression and metabolic analyses are needed to confirm the role of cytochrome P450, monooxygenase, and aminopeptidase in the metabolism of cyhalothrin resistance in strain S3-43^T^.

#### 3.4.4. Horizontal Gene Transfer Analysis

Genomic islands (GIs) are a prevalent form of horizontally transferred elements, typically comprising independent DNA fragments within prokaryotic chromosomes (Table S4). Based on the functions encoded by their genes, genomic islands can be categorized into virulence, resistance, metabolic, and symbiotic islands. Beyond several fundamental and homologous proteins that are functionally and structurally analogous to those found in other known strains, the genomic islands of S3-43^T^ encompass some non-homologous proteins capable of expressing additional functions. The existence of these non-homologous proteins may enable the strain to exhibit unique biological characteristics or adaptations, conferring it with enhanced viability or a competitive edge in a specific environment.

Most of the genes identified within the genomic islands of these twelve model strains were classified as putative genes or features with other movable genetic elements, such as insertion sequences and transposases. However, each strain carries genes related to enhanced adaptability, including genes encoding the secretion system (TSS), toxin antitoxin system, antibiotic resistance, and heavy metal transporters. Through genetic testing, we identified 22 GIs in S3-43^T^ containing 529 genes ranging from 5628 to 86,802 bp in length. Based on the functional analysis of GI genes, most of these known functional genes are involved in cellular metabolism and membrane transport functions. Annotation of the predicted gene functions of S3-43^T^ GIs revealed that some of these gene sequences could be expressed as DNA repair systems, such as the *ruvC*, *dnaE*, *vsr*, and *xerD*. Genes *ruvA*, *ruvB*, and *ruvC* can affect the effective survival rate of cells after radiation. The production of RuvA, RuvB, and RuvC proteins is required for the recombinational repair of UV-induced damage to DNA [92,93]. Genes *uvrA*, *uvrB*, *uvrC*, and *uvrD* are involved in the NER (nucleotide excision repair mechanism) repair pathway capable of dealing with a variety of potentially lethal DNA damage induced by radiation [94,95,96]. Gene *vsr* encodes a DNA mismatch endonuclease that can initiate VSP (Very Short Patch) mismatch repair [97,98], and is presumed t to be involved in radiation-induced DNA mismatches. Gene *osmC* plays an important role in peroxide metabolism and protects strains from oxidative stress [76,99].

## 4. Conclusions

This is the first study to describe the bacterial strain *Paracoccus qomolangaensis* S3-43^T^, isolated from the north slope of Mount Everest, and to investigate its mechanisms of radiation resistance and genomic function under extreme environmental stress. A variety of radiation-resistant antioxidant genes were identified, including *recR*, *recO*, *recA*, *radA,* and *ahpC*, among others. Therefore, we analyze that this is the reason why this strain was able to survive at high altitude in a cold-resistant, radiation-resistant, and oxidation-resistant environment. In addition, strain S3-43^T^ contained a number of cyhalothrin degradation-related genes, including cytochrome P450, monooxygenase, and aminopeptidase.

In conclusion, experimental and genomic analyses showed that strain S3-43^T^ possesses radiation and antioxidant resistance. A good antioxidant system gives it good resistance to ionizing radiation, while also exhibiting potential for pesticide degradation. Our results provide a foundation for investigating microbial radiation resistance and antioxidant mechanisms.

### Description of Paracoccus qomolangaensis sp. nov.

*Paracoccus qomolangaensis* (qomolangma. en’sis. N.L. masc. adj. qomolangmaensis about Mount Qomolangma, Tibet, China, where the type strain was isolated.)

Strain S3-43^T^ exhibits Gram-negative cells, with non-flagellated, ellipsoid (0.6–0.9 μm × 0.7–1.1 μm). Colonies on R2A are circular, convex, smooth, opaque, milky white, and approximately 2.0–3.0 mm in diameter after 72 h at 30 °C. It can grow at 10–35 °C (optimum 30 °C) and pH 6.0–11.0 (optimum pH 8.0), but it is intolerant of NaCl. Cells are positive for oxidase activity and reduction in nitrate, but urea, gelatin, starch, cellulose, Tweens 20, and 80 were not hydrolyzed. The strain is negative for production of H_2_S. In the API ZYM strip, it is positive for alkaline phosphatase, esterase (C4), leucine arylamidasem, naphthol-AS-BI-phosphohydrolase, and α-Glucosidase and negative for esterase lipase (C8), lipase (C14), acid phosphatase, N-acetyl-β-glucosaminidase, α-mannosidase, and β-fucosidase. Strain S3-43^T^ could not use L-Arabinose, D-Fructose, D-Glucose, D-Lactose, D-Galactose, D-Mannitol, D-Raffinose, D-Rhamnose, sucrose, or D-xylose as the sole carbon source. Meanwhile, strain S3-43^T^ could utilize L-Tyrosine as a unique nitrogen source but could not utilize L-Alanine, L-Aspartate, L-Histidine, and L-Glycine. Ubiquinone-10 (Q-10) is the sole respiratory quinone. The major polar lipids are diphosphatidylglycerol (DPG), four phospholipids (PL), an unidentified lipid (L), an unidentified aminolipid (AL), and an unidentified glycolipid (GL). The major cellular fatty acids (>10.0%) are summed feature 8 (comprising C_18:1_ ω7c and/or C_18:1_ ω6c).

The type strain S3-43^T^ (=KCTC 8297^T^ = GDMCC 1.3460^T^) was isolated from moraine samples being collected from the north slope of Mount Everest (28.02° N, 86.56° E), PR China. The DNA G+C content of the type strain is 67.2%. The GenBank/EMBL/DDBJ accession numbers for the genome and 16S rRNA gene sequences of type strain S3-43^T^ are CP119082 and ON527558, respectively.

## Figures and Tables

**Table 1:** List of genes encoding antioxidant DNA repair response proteins in the genomes of type strain S3-43^T^.

GenBank ID	Symbol	Protein Name	GenBank ID	Symbol	Protein Name
RS08485	*-*	cold-shock protein	RS02950	*yghU*	glutathione-dependent disulfide-bond oxidoreductase
RS14830	*-*	cold-shock protein	RS13140	*ruvB*	Holliday junction branch migration DNA helicase RuvB
RS13155	*ruvC*	crossover junction endodeoxyribonuclease RuvC	RS13150	*ruvA*	Holliday junction branch migration protein RuvA
RS06430	*-*	DNA alkylation repair protein	RS15020	*ruvX*	Holliday junction resolvase RuvX
RS01405	*recQ*	DNA helicase RecQ	RS00030	*-*	ligase-associated DNA damage response DEXH box helicase
RS10405	*vsr*	DNA mismatch endonuclease Vsr	RS00025	*pdeM*	ligase-associated DNA damage response endonuclease PdeM
RS03235	*mutL*	DNA mismatch repair endonuclease MutL	RS00040	*-*	ligase-associated DNA damage response exonuclease
RS01015	*mutS*	DNA mismatch repair protein MutS	RS15440	*msrB*	peptide-methionine (R)-S-oxide reductase MsrB
RS10955	*-*	DNA mismatch repair protein MutT	RS14820	*msrA*	peptide-methionine (S)-S-oxide reductase MsrA
RS07735	*rmuC*	DNA recombination protein RmuC	RS15445	*msrA*	peptide-methionine (S)-S-oxide reductase MsrA
RS12135	*radA*	DNA repair protein RadA	RS12420	*recA*	recombinase RecA
RS01075	*radC*	DNA repair protein RadC	RS08935	*recR*	recombination mediator RecR
RS04590	*recN*	DNA repair protein RecN	RS15945	*recJ*	single-stranded-DNA-specific exonuclease RecJ
RS13770	*recO*	DNA repair protein RecO	RS01565	*-*	UvrD-helicase domain-containing protein
RS00015	*recF*	DNA replication/repair protein RecF	RS08415	*-*	UvrD-helicase domain-containing protein
RS00950	*addA*	double-strand break repair helicase AddA	RS13235	*-*	UvrD-helicase domain-containing protein
RS00955	*addB*	double-strand break repair protein AddB	RS16030	*-*	NAD(P)H-quinone oxidoreductase
RS08490	*-*	DsbA family oxidoreductase	RS00945	*trxA*	thioredoxin
RS05580	*uvrA*	excinuclease ABC subunit UvrA	RS16155	*trxA*	thioredoxin
RS05645	*uvrB*	excinuclease ABC subunit UvrB	RS12075	*trxB*	thioredoxin-disulfide reductase
RS15085	*uvrC*	excinuclease ABC subunit UvrC			

**Table 2:** List of genes encoding pyrethroids degradation in the genomes of type strain S3-43^T^ and its related strains.

Gene Description	1	2	3	4	5	6	7	8	9	10	11	12
Cytochrome P450	**4**	3	4	3	4	3	2	2	2	4	3	4
Heme-degrading monooxygenase HmoA and related ABM domain proteins	**2**	1	2	2	2	0	2	2	2	2	2	2
Quinol monooxygenase YgiN	**1**	0	0	0	0	2	0	1	1	2	0	2
Aminopeptidase N, contains DUF3458 domain	**1**	1	1	1	1	1	1	1	1	1	1	1
D-aminopeptidase	**0**	0	0	1	1	0	0	0	0	0	0	0
L-aminopeptidase/D-esterase	**1**	1	1	2	2	1	1	1	1	1	1	1
Leucyl aminopeptidase	**2**	2	2	2	2	2	2	2	2	3	2	2
Methionine aminopeptidase	**2**	1	1	1	2	1	1	1	1	1	2	1
Putative aminopeptidase FrvX	**1**	0	1	1	1	0	0	0	0	1	0	0
Xaa-Pro aminopeptidase	**2**	4	4	3	2	2	2	1	3	2	3	3

Strains: 1. *Paracoccus qomolangmaensis* S3-43^T^, 2. *Paracoccus versutus* IAM 12814^T^, 3. *Paracoccus haematequi* LMG 30633^T^, 4. *Paracoccus acridae* KCTC 42932^T^, 5. *Paracoccus aerius* KCTC 42845^T^, 6. *Paracoccus zeaxanthinifaciens* ATCC 21588^T^, 7. *Paracoccus everestensis* S8-55^T^, 8. *Paracoccus subflavus* GY0581^T^, 9. *Paracoccus sediminis* CMB17^T^, 10. *Paracoccus lichenicola* YIM 132242^T^, 11. *Paracoccus fontiphilus* MVW-1^T^, and 12. *Paracoccus angustae* CCTCC AB 2015056^T^.

## Data Availability

The GenBank/EMBL/DDBJ accession numbers for the genome and 16S rRNA gene sequences of type strain S3-43^T^ are CP119082 and ON527558, respectively. The data can be accessed at https://www.ncbi.nlm.nih.gov/.

## Supplementary Materials

The following supporting information can be downloaded at: https://www.mdpi.com/article/10.3390/microorganisms13112441/s1, Figure S1: Neighbor-joining phylogenetic tree based on 16S rRNA gene sequences of the strain S3-43^T^ and the type strains of other closely related species in the genus *Paracoccus* and *Pikeienuella*, Figure S2: Minimum-evolution phylogenetic tree based on 16S rRNA gene sequences of the strain S3-43^T^ and the type strains of other closely related species in the genus *Paracoccus* and *Pikeienuella*, Figure S3: Maximum-likelihood phylogenetic tree based on 16S rRNA gene sequences of the strain S3-43^T^ and the type strains of other closely related species in the genus *Paracoccus* and *Pikeienuella*, Figure S4: Polar lipids profile of strain S3-43^T^, Figure S5: Genome comparisons of strain S3-43^T^ and their related reference strains, and Figure S6: Characteristic curves of the pan-genome and core genome of S3-43^T^; Table S1: Phenotypic characteristics of strain S3-43^T^ and its closely related type strains in the genus *Paracoccus*, Table S2: Whole cellular fatty acids composition of S3-43^T^ and the closely related type strains of the genus *Paracoccus*, Table S3: General genomic characteristics comparison of strain S3-43^T^ and its closely related species, and Table S4: Features of the GIs found in the genome of S3-43^T^. References [100,101,102,103,104] are cited in the supplementary materials.
