# Supplementary material for: Comparative Genomics and Phylogenomics of Novel Radiation-Resistant Bacterium Paracoccus qomolangmaensis sp. nov. S3-43T, Showing Pyrethroid Degradation
Source: Microorganisms. 2025 Oct 24;13(11):2441. doi: 10.3390/microorganisms13112441 (PMC12654122; doi:10.3390/microorganisms13112441)
Supplement: Supplementary file 1 [file microorganisms-13-02441-s001.zip › microorganisms-3890312-supplementary.pdf]

Supplementary Materials

Table S1-S4

Table S1. Phenotypic characteristics of strain S3-43^T and its closely related type strains in the genus *Paracoccus*.

Strains: 1. *Paracoccus qomolangmaensis* S3-43^T; 2. *Paracoccus haematequi* LMG 30633^T; 3. *Paracoccus acridae* KCTC 42932^T; 4. *Paracoccus aerius* KCTC 42845^T; 5. *Paracoccus angustae* CCTCC AB 2015056^T; 6. *Paracoccus fontiphilus* MVW-1^T; 7. *Paracoccus zeaxanthinifaciens* ATCC 21588^T; 8. *Paracoccus everestensis* S8-55^T; 9. *Paracoccus lichenicola* YIM 132242^T; 10. *Paracoccus sediminis* CMB17^T; 11. *Paracoccus subflavus* GY0581^T; 12. *Paracoccus versutus* IAM 12814^T. All related type strains of S3-43^T were positive for Oxidase activity, Alkaline phosphatase, Leucine arylamidase. All data were from this study. +, Positive; -, negative; w, weakly positive.

**Characteristics**	**1**	**2**	**3**	**4**	**5**	**6***	**7***	**8***	**9***	**10***	**11***	**12***
**Isolation source**	**Moraine**	Horse blood	Insect	Air	Soil	Freshwater	Coast	Moraine	Lichen	Sediment	Sediment	Soil
**Colony Color**	**Milky white**	Slightly Orange	Orange-red	Orange	Orangey pink	Light Orange	Yellow to orange	Orange	Deep orange	Orange	Light yellow	Brownish yellow
**Growth temperature (℃) range (optium)**	**10-35 (30)**	10-45 (30)	10-40 (25-30)	4-41 (30)	4-45 (28-37)	15-30 (20-25)	10-40 (25-40)	10-37 (30)	10-39 (28-35)	4-40 (25-30)	15-40 (35-37)	17-40 (30-37)
**pH range (optium)**	**6-11(8)**	5-7.5(5)	5-10（6.5-7.5）	4.5-10(8)	6-9(6-7)	6-9(6-7)	6.1-9.1	7-11(8)	4-10(7-8)	5.5-9(6.5-7)	6-10.5(7-8)	6.5-9.5(7.5-8)
**NaCl tolerance range (optium) (%, w/v)**	**0-6(1-2)**	0-1(1)	0-4(0-1)	0-10(0-2)	0-5(0-1)	0-8(0-3)	3-8	0	0-8(0-2)	0-11(0.5-1)	0-6(3.5)	ND
**Oxidase activity**	**+**	+	+	+	+	+	+	+	+	+	+	+
**Production of H_2S**	**-**	-	-	-	+	ND	ND	-	-	ND	ND	ND
**Reduction of nitrate**	**+**	-	-	-	-	-	-	-	-	+	-	+
**Hydrolysis of:**												
Urea	**-**	-	-	-	-	ND	+	-	+	-	ND	ND
Tween 20	**-**	-	-	-	-	-	ND	-	+	-	ND	ND
Tween 80	**-**	-	+	+	-	+	-	-	+	-	ND	ND
Gelatin	**-**	-	-	+	-	-	-	-	-	-	ND	ND
Starch	-	-	+	+	-	-	-	-	-	-	ND	ND
Cellulose	-	-	-	-	-	-	ND	-	-	ND	ND	ND
**Utilization as carbon sources**												
L-Arabinose	**-**	+	+	+	+	ND	-	+	ND	+	ND	+
D-Fructose	**-**	+	+	+	+	ND	ND	+	+	ND	ND	+
D-Glucose	**-**	+	+	w	+	ND	+	-	+	+	-	+
D-Lactose	**-**	-	+	w	+	ND	+	-	ND	ND	ND	ND
D-Galactose	**-**	+	-	+	-	ND	+	-	+	ND	ND	+
D-Mannitol	**-**	+	-	+	+	ND	+	w	+	+	ND	+
D-Raffinose	**-**	-	-	w	w	ND	-	w	+	ND	ND	ND
D-Rhamnose	**-**	-	+	+	+	ND	-	-	+	ND	ND	ND
Sucrose	**-**	-	-	-	+	ND	ND	-	ND	ND	ND	+
D-xylose	**-**	+	-	-	-	ND	ND	-	+	ND	ND	ND
**Utilization as**												

**nitrogen sources**												
L-Alanine	**-**	-	-	-	-	ND	-	+	ND	ND	ND	ND
L-Aspartate	**-**	-	-	-	-	ND	+	+	ND	ND	ND	+
L-Histidine	**-**	-	-	-	+	ND	-	+	ND	ND	ND	+
L-Tyrosine	**+**	+	+	-	+	ND	ND	+	ND	ND	ND	ND
L-Cysteine	**-**	-	-	+	-	ND	ND	-	ND	ND	ND	-
**Enzymatic activity**												
Alkaline phosphatase	**w**	+	+	w	+	+	ND	+	+	+	+	ND
Esterase (C4)	**+**	+	+	+	+	+	ND	+	+	+	+	ND
Lip esterase (C8)	**-**	+	+	+	+	+	ND	+	+	+	+	ND
Lipase (C14)	**-**	-	-	-	-	ND	ND	-	ND	-	ND	ND
Acid phosphatase	**-**	-	-	w	+	+	ND	+	+	+	+	ND
Leucine arylamidase	**+**	+	+	+	+	+	ND	+	+	+	+	ND
Naphthol-AS-BI-phosphate hydrolase	**w**	+	+	w	+	+	ND	-	+	+	+	ND
N-acetyl-β-glucosaminase	**-**	-	-	-	-	ND	ND	-	ND	-	ND	ND
α-Mannosidase	**-**	-	-	-	-	ND	ND	-	ND	-	ND	ND
α-Glucosidase	**+**	+	-	+	+	+	ND	ND	+	-	--	ND
β-Fucosidase	**-**	-	-	-	-	ND	ND	-	ND	-	ND	ND
**Motility**	**-**	-	-	+	+	-	-	-	-	-	-	+
**Spore formation**	**-**	-	-	-	-	-	-	-	-	-	-	-
**Major fatty acids***	**$C_{18:1}$ *ω7c*, $C_{18:0}$**	$C_{18:1}$ *ω*7c, $C_{18:0}$, $C_{17:0}$	$C_{18:1}$ *ω7c*, $C_{16:0}$, $C_{18:0}$, $C_{10:0}$ 3-OH	$C_{18:1}$ *ω7c*, $C_{18:0}$	$C_{18:1}$ *ω7c*, $C_{18:0}$,	$C_{18:1}$ *ω7c*, $C_{18:0}$	$C_{18:1}$ *ω*7c	$C_{18:1}$ ω7c and $C_{18:1}$ ω6c	$C_{18:1}$ *ω*7c	$C_{18:1}$ *ω*7c	$C_{18:1}$ *ω*7c	ND
**DNA G+C content (mol%)**	**67.2**	66.6	60.6	63.5	68.1	63.4	67.6	64.3	67.1	62.2	65.6	67.5

Data were taken from: 6, Sheu, S. Y. *et al.* 2018, [100]; 7*, Berry, A. *et al.* 2003, [101]; 8*, Cui, X. *et al*. 2022, [6]; 9*, Lang, L. *et al.* 2021, [102]; 10*, Pan, J. *et al.* 2014, [103]; 11*, Zhang, G. *et al.* 2019, [104]; 12*, Katayama, Y. *et al.* 1995, [11].

Table S2. Whole cellular fatty acids composition of S3-43^T and the closely related type strains of the genus *Paracoccus*.

Strains: 1. *Paracoccus qomolangmaensis* S3-43^T; 2. *Paracoccus haematequi* LMG 30633^T; 3. *Paracoccus acridae* KCTC 42932^T; 4. *Paracoccus aerius* KCTC 42845^T; 5. *Paracoccus angustae* CCTCC AB 2015056^T; 6. *Paracoccus fontiphilus* MVW-1^T; 7. *Paracoccus zeaxanthinifaciens* ATCC 21588^T; 8. *Paracoccus everestensis* S8-55^T; 9. *Paracoccus lichenicola* YIM 132242^T; 10. *Paracoccus sediminis* CMB17^T; 11. *Paracoccus subflavus* GY0581^T; 12. *Paracoccus versutus* IAM 12814^T. Only fatty acids percentages amounting to 0.5% or higher were shown. L (<0.5%); ND, not detected.

**Fatty acids (%)**	**1**	**2**	**3**	**4**	**5**	**6***	**7***	**8***	**9***	**10***	**11***	**12***
**Saturated fatty acids**												
**$C_{16:0}$**	**1.5**	1.9	10.6	ND	ND	1.8	L	0.7	3.9	L	2.2	ND
**$C_{17:0}$**	**1.0**	6.5	ND	3.0	ND	3.6	L	0.6	2.9	0.6	L	ND
**$C_{18:0}$**	**6.3**	8.7	5.0	5.9	6.1	10.0	3.6	5.4	6.5	4.6	9.4	ND
**Hydroxy fatty acids**												
**$C_{10:0}$ 3-OH**	**2.9**	ND	10.2	ND	3.7	2.4	4.9	4.0	1.3	2.1	2.1	ND
**$C_{18:0}$ 3-OH**	**2.1**	1.4	ND	ND	1.1	1.9	0.7	L	2.9	ND	2.2	ND
**Sum In Feature 2**	**2.6**	2.7	ND	2.0	2.8	1.9	2.9	3.76	L	2.9	2.3	ND
**Summed Feature 8**	**83.3**	74.9	70.4	84.5	79.7	75.9	80.2	78.2	77.6	87.6	79.5	ND

Summed features represent groups of two fatty acids which could not be separated by GLC and the MIDI system. Summed feature 2 contained 12:0 aldehyde and/or unknown 10.9525, and summed feature 8 contained 18:1 *ω6c* and /or 18:1 *ω7c*.

Data were taken from: 6, Sheu, S. Y. *et al.* 2018, [100]; 7*, Berry, A. *et al.* 2003, [101]; 8*, Cui, X. *et al*. 2022, [6]; 9*, Lang, L. *et al.* 2021, [102]; 10*, Pan, J. *et al.* 2014, [103]; 11*, Zhang, G. *et al.* 2019, [104]; 12*, Katayama, Y. *et al.* 1995, [11].

Table S3. General genomic characteristics comparison of strain S3-43^T and its closely related species.

Strains: 1. *Paracoccus qomolangmaensis* S3-43^T; 2. *Paracoccus haematequi* LMG 30633^T; 3. *Paracoccus acridae* KCTC 42932^T; 4. *Paracoccus aerius* KCTC 42845^T; 5. *Paracoccus angustae* CCTCC AB 2015056^T; 6. *Paracoccus fontiphilus* MVW-1^T; 7. *Paracoccus zeaxanthinifaciens* ATCC 21588^T; 8. *Paracoccus everestensis* S8-55^T; 9. *Paracoccus lichenicola* YIM 132242^T; 10. *Paracoccus sediminis* CMB17^T; 11. *Paracoccus subflavus* GY0581^T; 12. *Paracoccus versutus* DSM 17099^T.

**Characteristics**	**1**	**2**	**3**	**4**	**5**	**6***	**7***	**8***	**9***	**10***	**11***	**12***
**Genome size (bp)**	**3680447**	4103082	3989817	4166940	4569394	4106270	3049695	4230436	3707728	3512108	3183584	5500748
**Contig N50**	**3221824**	120146	269428	172164	164745	51233	237387	2957008	257193	246067	284404	78673
**CDS**	**3746**	4119	3935	4111	4466	4103	2942	4094	3590	3427	3130	5137
**RNA**	**52**	50	3879	50	51	50	54	59	48	52	49	62
**rRNA**	**6**	3	3	3	3	3	3	9	3	3	3	9
**tRNA**	**46**	47	49	47	48	47	51	50	45	49	46	53
**5S rRNA**	**2**	1	1	1	1	1	2	3	1	1	1	3
**16S rRNA**	**2**	1	1	1	1	1	1	3	1	1	1	3
**23S rRNA**	**2**	1	1	1	1	1	0	3	1	1	1	3
**KEGG**	**2031**	2180	2208	2245	2419	2197	1899	2179	2068	1948	1846	3041
**COG**	**3345**	3117	3143	3237	3478	3169	2539	3736	2879	2758	2554	4377
**NR**	**3595**	4100	3880	3955	4164	3994	2912	4023	3271	3421	3100	5471
**Pfam**	**3076**	3339	3345	3451	3703	3419	2660	3471	3104	2923	2687	4694
**Swiss-Prot**	**2613**	1608	1681	1681	1746	1641	1367	3038	1511	1432	1360	2082
**CAZyme**	**73**	143	135	145	154	158	130	119	154	45	123	144
**Secondary Metabolite**	**3**	7	4	4	9	9	9	5	10	4	5	6
**DNA G+C content (mol%)**	**67.2**	66.6	65.3	65.0	66.5	66.2	67.6	64.3	67.1	62.2	65.6	67.4
**GenBank accession number**	**GCA_029027965.1**	GCA_900631945.1	GCA_014642735.1	GCA_016757275.1	GWHBJVS00000000	GCA_017356265.1	GCA_000420145.1	GCA_021491915.1	GCA_009708075.1	GCA_900188295.1	GCA_004310345.1	GCA_029909795.1

Data were taken from: 6, Sheu, S. Y. *et al.* 2018, [100]; 7*, Berry, A. *et al.* 2003, [101]; 8*, Cui, X. *et al.* 2022, [6]; 9*, Lang, L. *et al.* 2021, [102]; 10*, Pan, J. *et al.* 2014, [103]; 11*, Zhang, G. *et al.* 2019, [104]; 12*, Katayama, Y. *et al.* 1995, [11].

Table S4. Features of the GIs found in the genome of S3-43^T

GI	Length (bp)	Total no. of gene	Hypothetical proteins	Predicted function
1	86802	90	34	Post-translational modification, protein turnover, chaperones; Transcription; Inorganic ion transport and metabolism; Cell wall/membrane/envelope biogenesis; Energy production and conversion; Replication, recombination and repair; Defense mechanisms
2	11854	14	7	Transcription; Replication, recombination and repair; Defense mechanisms
3	77224	84	30	Replication, recombination and repair; Inorganic ion transport and metabolism; Transcription; Cell wall/membrane/envelope biogenesis; Intracellular trafficking, secretion, and vesicular transport; Carbohydrate transport and metabolism; Energy production and conversion; Nucleotide transport and metabolism; Post-translational modification, protein turnover, chaperones
4	9118	9	4	Replication, recombination and repair; Nucleotide transport and metabolism
5	7140	7	4	Replication, recombination and repair
6	49952	56	42	Amino acid transport and metabolism; Nucleotide transport and metabolism; Replication, recombination and repair; Transcription; Inorganic ion transport and metabolism; Cell wall/membrane/envelope biogenesis; Post-translational modification, protein turnover, chaperones
7	10081	11	4	Lipid transport and metabolism; Replication, recombination and repair
8	53520	57	37	Transcription; Replication, recombination and repair; Cell wall/membrane/envelope biogenesis; Inorganic ion transport and metabolism; Amino acid transport and metabolism; Secondary metabolites biosynthesis, transport and catabolism; Energy production and conversion
9	13831	17	7	Replication, recombination and repair
10	7769	10	6	Replication, recombination and repair; Cell wall/membrane/envelope biogenesis
11	16487	13	2	Replication, recombination and repair; Transcription
12	10897	15	8	Replication, recombination and repair; Transcription; Translation, ribosomal structure and biogenesis; Cell wall/membrane/envelope biogenesis
13	10726	12	4	Replication, recombination and repair; Defense mechanisms
14	5628	11	6	Replication, recombination and repair; Transcription
15	7748	10	8	Inorganic ion transport and metabolism; Coenzyme transport and metabolism; Replication, recombination and repair
16	28941	37	35	Intracellular trafficking, secretion, and vesicular transport; Transcription; Translation, ribosomal structure and biogenesis; Replication, recombination and repair
17	7644	10	7	Energy production and conversion; Replication, recombination and repair; Coenzyme transport and metabolism
18	7212	7	0	
19	9597	10	7	Inorganic ion transport and metabolism; Energy production and conversion; Coenzyme transport and metabolism; Carbohydrate transport and metabolism
20	24091	25	11	Amino acid transport and metabolism; Replication, recombination and repair; Intracellular trafficking, secretion, and vesicular transport; Transcription; Nucleotide transport and metabolism
21	12520	10	10	Inorganic ion transport and metabolism; Transcription; Nucleotide transport and metabolism; Lipid transport and metabolism; Replication, recombination and repair; Post-translational modification, protein turnover, chaperones
22	17897	14	7	Cell wall/membrane/envelope biogenesis; Inorganic ion transport and metabolism; Post-translational modification, protein turnover, chaperones
**Total**	**486679**	**529**	**280**	

Fifure S1-S6

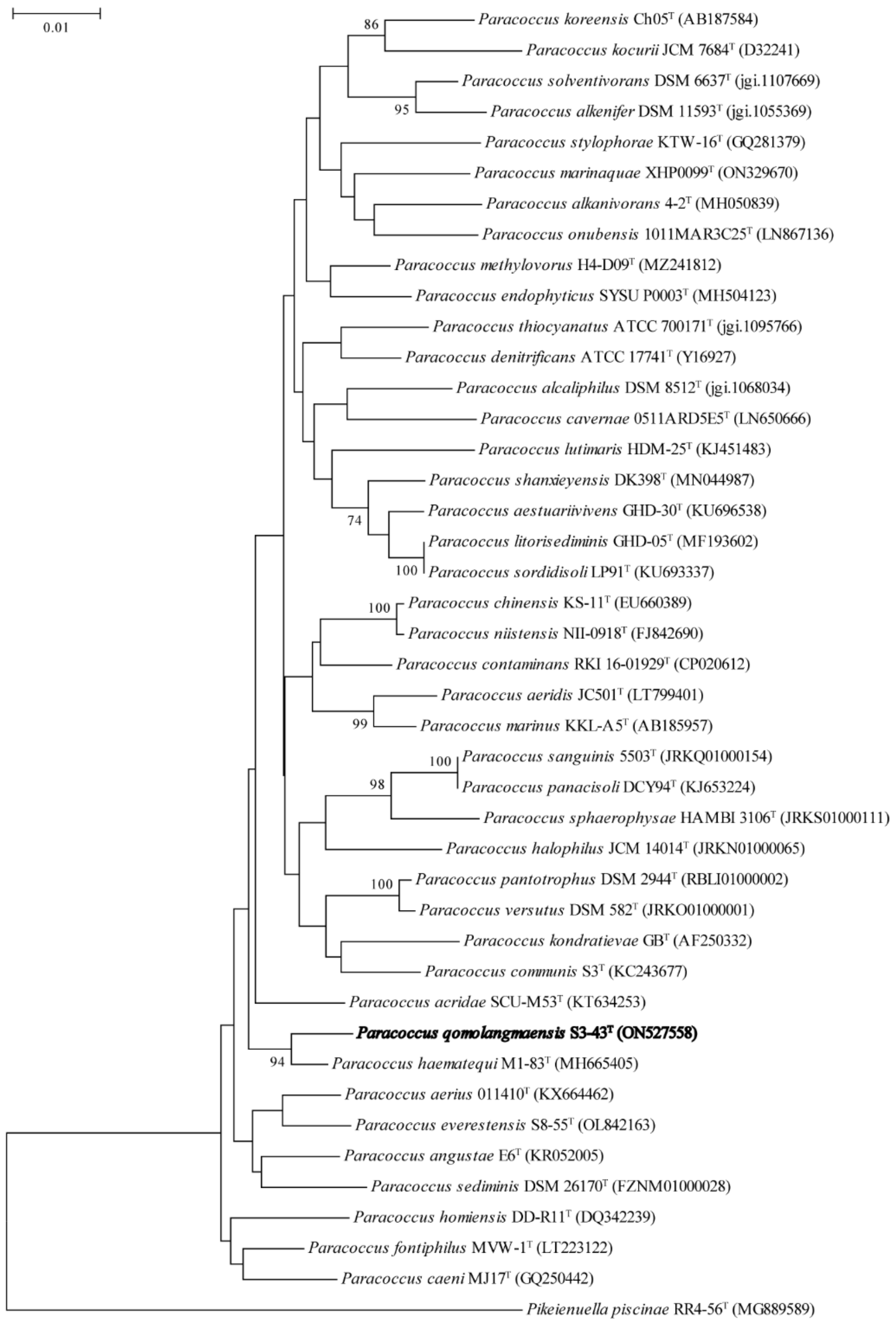

Figure S1. Neighbor-joining phylogenetic tree based on 16S rRNA gene sequences of the strain S3-43^{T} and the type strains of other closely related species in the genus *Paracoccus* and *Pikeienuella*. *Pikeienuella piscinae* RR4-56^{T} (MG889589) was used as an outgroup. The numbers on the tree indicate the percentages of bootstrap sampling derived from 1000 replications and the bootstrap values higher than 70% are shown. Bar, 0.01 substitutions per nucleotide position.

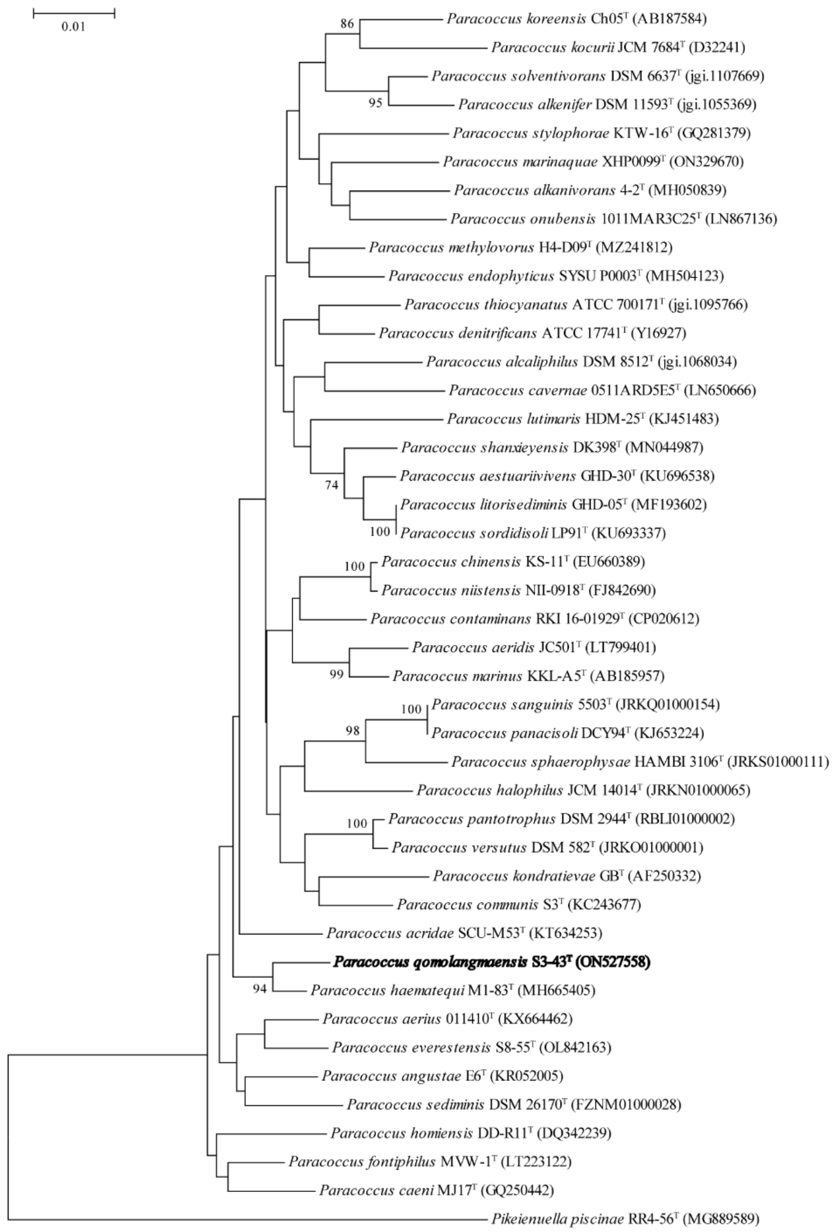

Figure S2. Minimum-evolution phylogenetic tree based on 16S rRNA gene sequences of the strain S3-43^T, and the type strains of other closely related species in the genus *Paracoccus* and *Pikeienuella*. *Pikeienuella piscinae* RR4-56^T (MG889589) was used as an outgroup. The numbers on the tree indicate the percentages of bootstrap sampling derived from 1000 replications and the bootstrap values higher than 70% are shown. Bar, 0.01 substitutions per nucleotide position.

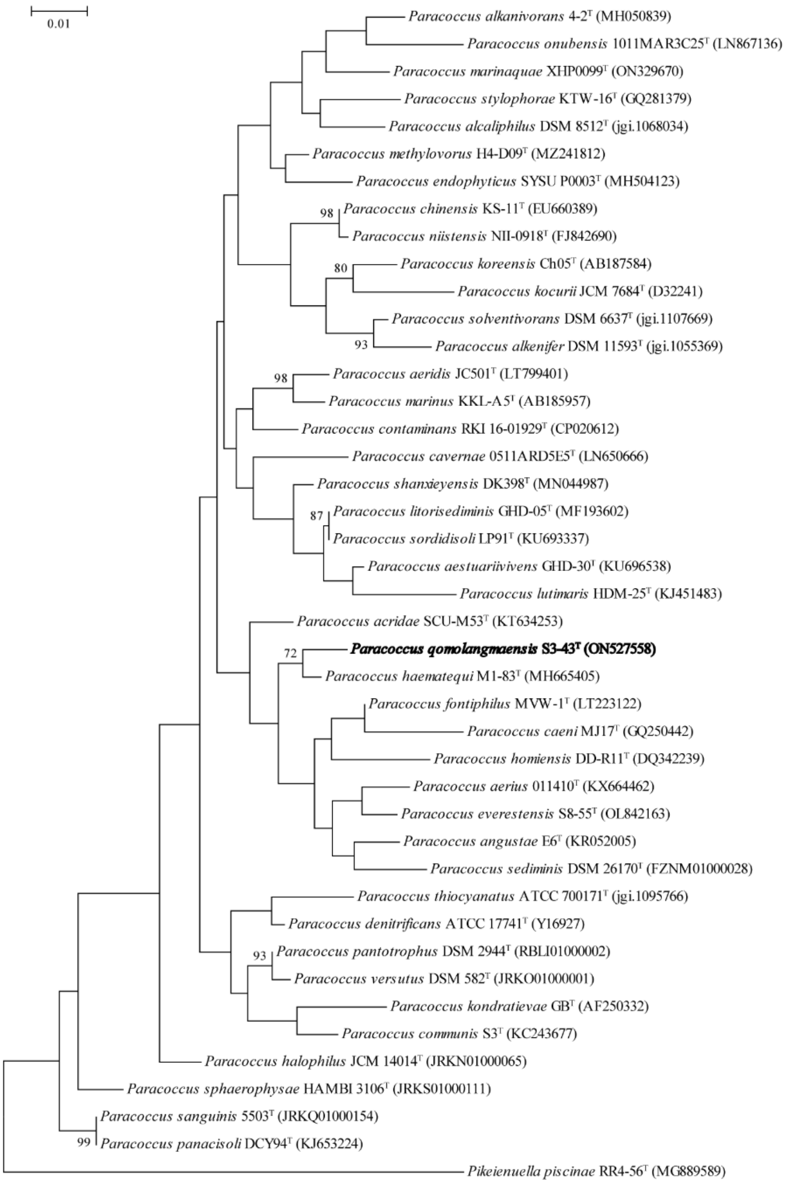

Figure S3. Maximum-likelihood phylogenetic tree based on 16S rRNA gene sequences of the strain S3-43^{T}, and the type strains of other closely related species in the genus *Paracoccus* and *Pikeienuella*. *Pikeienuella piscinae* RR4-56^{T} (MG889589) was used as an outgroup. The numbers on the tree indicate the percentages of bootstrap sampling derived from 1000 replications and the bootstrap values higher than 70% are shown. Bar, 0.01 substitutions per nucleotide position.

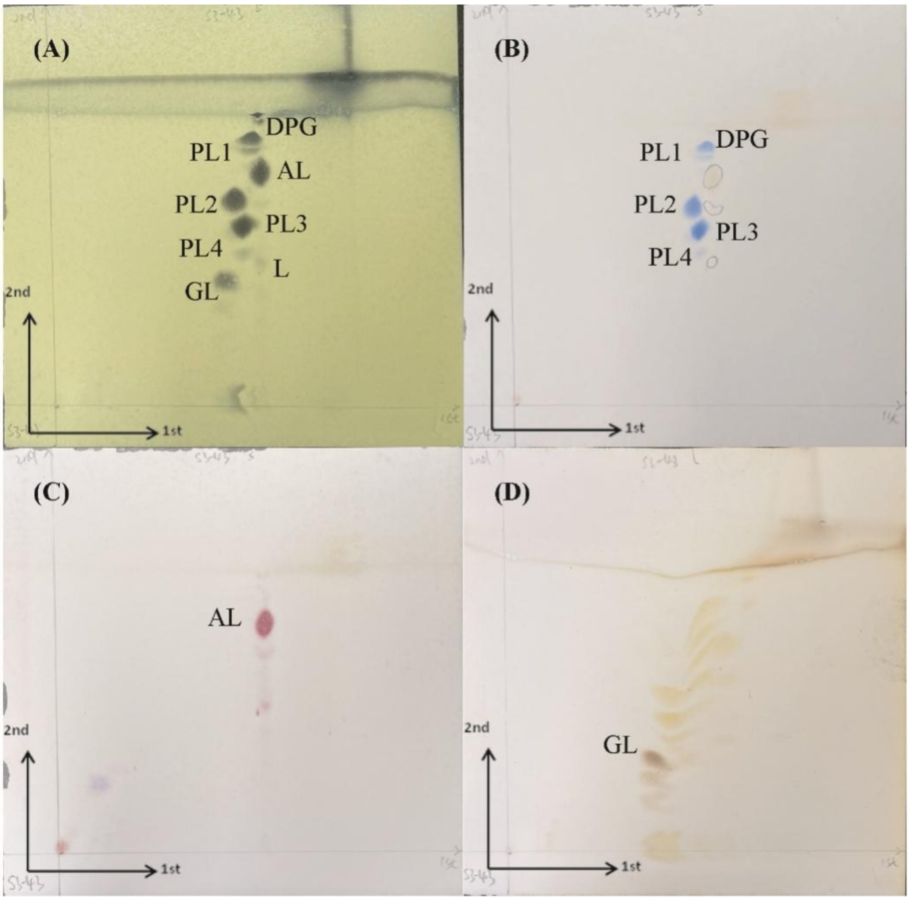

Figure S4. Polar lipids profile of strain S3-43^{T}. Total lipids were visualized after two-dimensional TLC and applying 5% ethanolic molybdatophosphoric acid. The solvent system was phosphomolybdic acid (**A**), molybdenum blue (**B**), indigohydrone (**C**), and α-naphthol (**D**) from left to right and top to bottom.

DPG, Diphosphatidylglycerol; PL1-4, Phospholipids; L, unidentified lipid; AL, unidentified aminolipid; GL, unidentified glycolipid

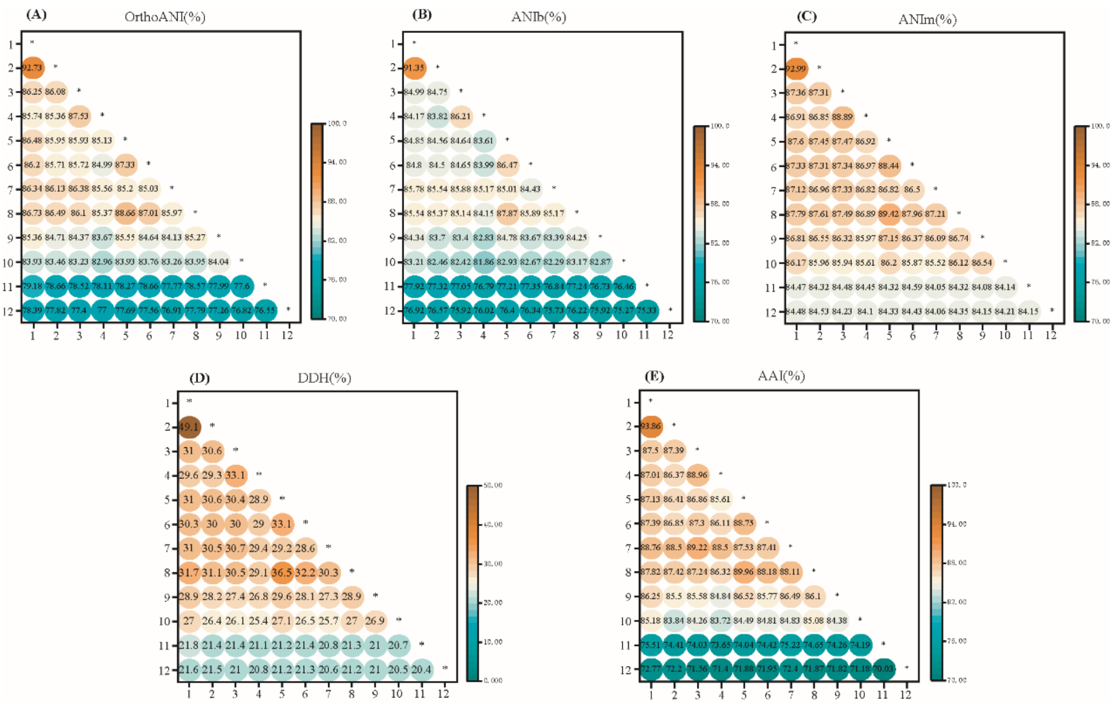

Figure S5. Genome comparisons of strain S3-43^{T} and their related reference strains including OrthoANI value (A), ANIb value (B), ANIm value (C), dDDH value (D), and AAI value (E). 1-12 represented S3-43^{T}, *P.haematequi* LMG 30633^{T}, *P. acridae* KCTC 42932^{T}, *P. aerius* KCTC 42845^{T}, *P.angustae* CCTCC AB 2015056^{T}, *P. fontiphilus* MVW-1^{T}, *P. everestensis* S8-55^{T}, *P. lichenicola* YIM 132242^{T}, *P. sediminis* CMB17^{T}, *P. subflavus* GY0581^{T}, *P. zeaxanthinifaciens* ATCC 21588^{T}, *P. versutus* DSM 17099 ^{T}.

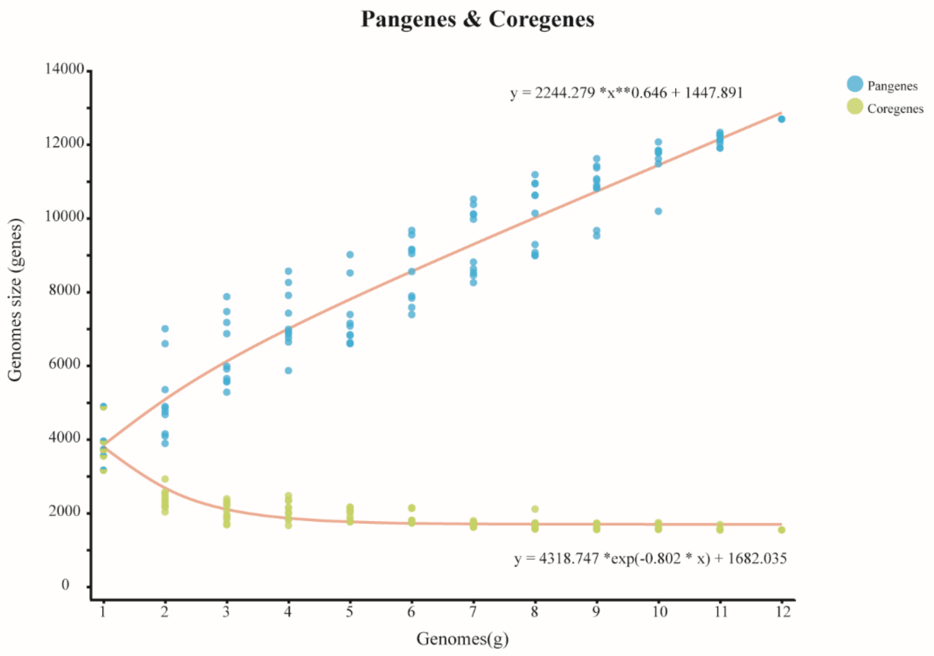

Figure S6. Characteristic curves of the pan-genome and core genome of S3-43^{T}
